# The Burden of Cervical Cancer in Korea: A Population-Based Study

**DOI:** 10.3390/ijerph17176308

**Published:** 2020-08-30

**Authors:** Jinhee Kim, Donghwan Lee, Kyung-Bok Son, SeungJin Bae

**Affiliations:** 1 College of Pharmacy, Ewha Womans University, Seoul 03760, Korea; kimjin7247@naver.com (J.K.); sonkyungbok@ewha.ac.kr (K.-B.S.); 2 Department of Statistics, Ewha Womans University, Seoul 03760, Korea; donghwan.lee@ewha.ac.kr

**Keywords:** cervical cancer, carcinoma in situ, incidence, cost, burden of disease, HPV

## Abstract

This study used the Korean National Health Insurance (NHI) claims database from 2011 to 2017 to estimate the incidence and the incidence-based cost of cervical cancer and carcinoma in situ of cervix uteri (CIS) in Korea. The primary outcome was the direct medical cost per patient not diagnosed with cervical cancer (C53) or CIS (D06) 2 years prior to the index date in the first year after diagnosis. A regression analysis was conducted to adjust for relevant covariates. The incidence of cervical cancer tended to decrease from 2013 to 2016, while that of CIS increased. In particular, the incidence rate of CIS in women in their 20 s and 30 s increased by 56.8% and 28.4%, respectively, from 2013 to 2016. The incidence-based cost of cervical cancer and CIS was USD 13,058 and USD 2695 in 2016, respectively, which increased from 2013. Multivariate regression analysis suggested that age was the most influential variable of the cost in both patient groups, and the cost was highest in those aged over 60, i.e., the medical cost was significantly lower in younger women than their older counterparts. These findings suggest that targeting younger women in cervical cancer prevention is a reasonable option from both economic and public health perspectives.

## 1. Introduction

Cervical cancer is the fourth most widespread cancer, with an estimated 569,800 new cases in 2018. It is one of the leading causes of female death worldwide, with 80% of all deaths occurring in Africa and Southwest Asia [1]. In Korea, the incidence rate of cervical cancer in 2016 was 10.8 per 100,000 women. Cervical cancer remains the seventh most common cancer in the Korean population [2].

Unlike other cancers, cervical cancer is preventable because it takes several years to develop, which allows detection at early stages of the disease [3]. Cervical cancer is a sexually transmitted disease (STD) caused by human papillomavirus (HPV) infection. Known risk factors for cervical cancer include young age at first sexual intercourse, smoking, and low economic status [4]. Continuous infection with HPV can lead to abnormal proliferation of the cervical epithelium and development of cervical intraepithelial neoplasia (CIN). CIN is categorized into CIN1 (mild), CIN2 (moderate), and CIN3 (severe) subtypes depending on the proportion of the thickness of the abnormal epithelium cells [5]. Most mild stages of CINs spontaneously regress. However, some cases progress to higher grades, such as carcinoma in situ of cervix uteri (CIS). CIS is a precancerous lesion that is called stage 0 cervical cancer [6]. A previous study found that 24–75% of CIS cases progressed to invasive cervical cancer if untreated [7]. In contrast, this precancerous lesion has nearly a 100% 5 year survival rate when it is detected and treated early [6].

Cervical screening primarily seeks to detect and remove CIN or precancerous lesions (such as CIS) in order to reduce the incidence of cervical cancer. This screening also aims to reduce disease progression through early cancer detection [8]. Precancerous progression, in particular, does not cause specific symptoms. Therefore, these lesions are not detected unless a patient undergoes screening [9]. For this reason, cervical cancer screening has been introduced in many countries. Cervical cancer screening was introduced in Korea in 1999 as a part of the National Cancer Screening Program (NCSP) [10]. Routine cervical cancer screening has led to decreased incidence and cancer-specific mortality in Northern and Central Europe, as well as in North America [9]. However, in Korea, despite a downtrend in the incidence of cervical cancer in recent years, the slope of decline is marginal (annual percentage change (APC) from 1999 to 2016 is −3.6%; [11]). However, the age-standardized rates (ASRs) of CIS have increased significantly from 7.5 per 100,000 patients in 1993 to 19.0 per 100,000 patients in 2009. The trends of increasing precancerous disease are observed not only in Korea, but also in the United States and the Netherlands [12,13]. In addition, a previous study using the Korean Central Cancer Registry (KCCR) data showed a strong positive correlation between the incidence of CIS and the cervical cancer screening rate [14].

Although the incidence of cervical cancer in Korea has decreased, it remains similar to the international incidence rate (13.1 per 100,000), including that in developing countries [15]. The incidence of cervical cancer in Korea is also higher than that in other developed countries, such as the United States and the United Kingdom [10]. In 2018, the World Health Organization (WHO) proposed a global strategy of lowering the incidence rate of cervical cancer to less than 4 per 100,000 [16]. In Korea, active efforts are needed to further reduce the incidence of cervical cancer and CIS. Therefore, estimating the current economic burden of cervical cancer and CIS in Korean society can be the basis for establishing effective prevention policies.

Several prior studies have assessed the cost of cervical cancer in Korea, although most are prevalence-based studies [17,18]. One previous incidence-based study suffered various methodological limitations, including: the risk of overestimation due to the study design, cost that was not adjusted with the consumer price index (CPI), and failure to consider factors that can affect the cost [19]. In addition, there is no recent study. Therefore, the purpose of our study was to estimate the incidence rate and incidence-based cost of precancerous lesions and cervical cancer using up-to-date data from 2013 to 2016 in Korea

## 2. Materials and Methods

### 2.1. Database

We used Korean National Health Insurance (NHI) claims data from the Health Insurance Review and Assessment (HIRA), which covers almost 98% of the total population (of approximately 50 million) in Korea [20]. The Korean NHI is mostly reimbursed through a fee-for-service scheme that includes 7 conditions that are reimbursed with the diagnosis related group (DRG). A vast majority (99%) of the claims data is generated electronically in the process of reimbursement [21]. The NHI claims data consist of patients’ general information and health care services, including inpatient and outpatient prescriptions, and diagnosis information. The general information contains patients’ demographic characteristics and personal identification codes. The information on health care services includes the diagnosis code, procedure codes, claim dates, and cost information (patient out-of-pocket costs and payer costs) [22]. We used data from January 2011 to December 2017. This study was approved by the Institutional Review Board of Ewha Womans University (IRB File No. 168–10).

### 2.2. Study Population

This is a retrospective, population-based study. The study population consisted of Korean women over 20 years old based on previous studies [14,19]. We defined newly diagnosed cervical cancer and CIS patients to evaluate the incidence-based costs. We defined new patients who were diagnosed with the corresponding diagnosis codes, the International Classification of Diseases (ICD) 10th Revision of cervical cancer (C53) and CIS (D06) from 2013 to 2016 based on the primary and secondary diagnosis [18].

**Figure 1:**
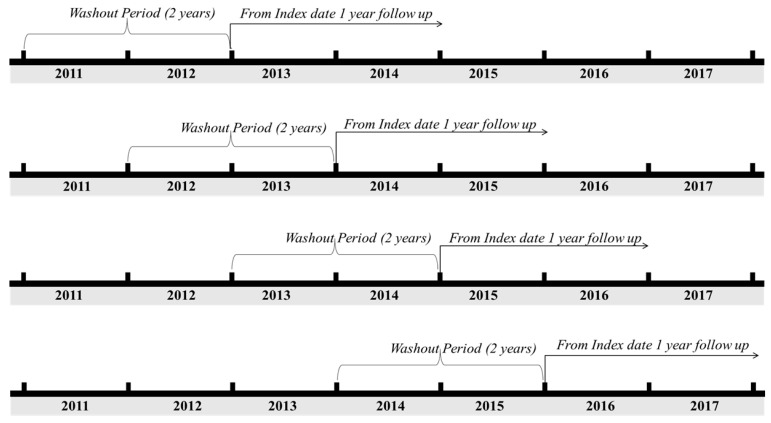
Study design of operational definition of new patients between 2013 and 2016.

The washout period was defined as 2 years based on the clinician’s advice that each patient can be defined as a new patient if there was no diagnosis of the same disease in the preceding two years from the index date (which was that of the first diagnosis according to the corresponding ICD codes) from 2013 to 2016. In addition, to improve the accuracy of cancer diagnosis, patients with the following specific classification codes were included: V027, V193, and V194, which represent cancer-specific deductible insurance codes in Korea [23]. The follow-up period was defined as 1 year from the index date to estimate the annual cost per patient of treatment. The study design is shown in Figure 1.

The Charlson comorbidity index (CCI) was used to adjust for the patient’s comorbidity, which was measured 1 year prior to the index date [24,25]. The main outcome was the incidence-based annual medical cost per cervical cancer patient and CIS patient. The incidence-based cost was estimated in terms of the total healthcare cost, including hospitalizations, outpatient visits, and prescription drugs, for 1 year after the index date. All costs were adjusted by the 2016 medical care component of the CPI [26], and converted using the average exchange rate from 2013 to 2016 (1 USD = 1110 KRW) [27].

### 2.3. Statistical Analysis

We used a parametric test (Analysis of Variance, ANOVA) and nonparametric test (Kruskal–Wallis test) to compare the medical cost differences by age group. Regression analysis was used to adjust for other variables affecting medical costs. Independent variables were categorized as age group (20–39 years = 0, 40–59 years = 1, 60+ = 2), year (2013 = 0, 2014 = 1, 2015 = 2, 2016 = 3), and CCI score. The distribution of medical cost was right-skewed; therefore, the log-transformation conversion was considered [28]. However, despite the logarithmic transformation, the normality test failed (*p* < 0.05). Therefore, in addition to the linear regression model (LRM), generalized linear models (GLMs) were used. A gamma distribution was assumed to distribute the medical costs. GLMs are often used to analyze irregular data [29]. In the regression analysis, univariate analysis was performed to evaluate the effect of each independent variable. Multivariate analysis was performed to adjust for confounding factors, such as age, year, and CCI. After analysis, a model fit was compared based on the Akaike information criterion (AIC). All analyses were conducted using SAS software (version 9.4, SAS Institute Inc., Cary, NC, USA). The significance level was *p* < 0.05.

## 3. Results

### 3.1. Ovreall

**Figure A1:**
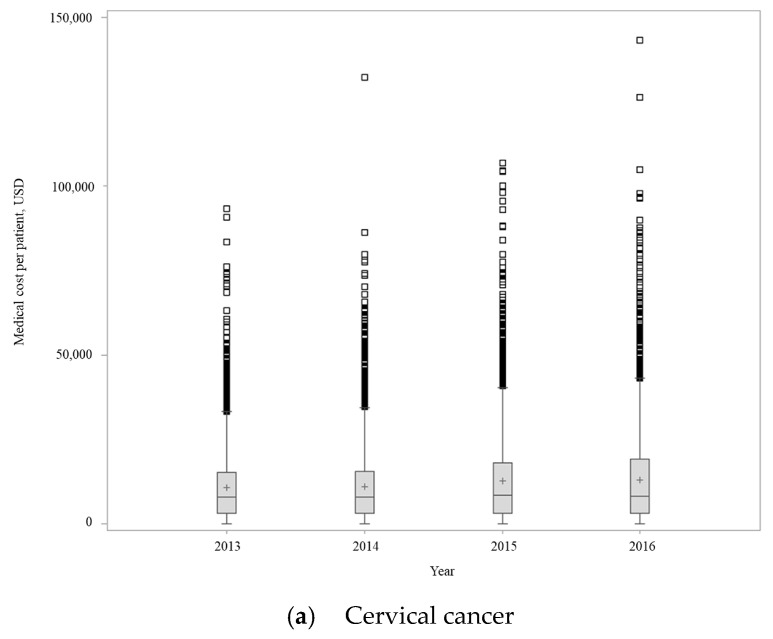

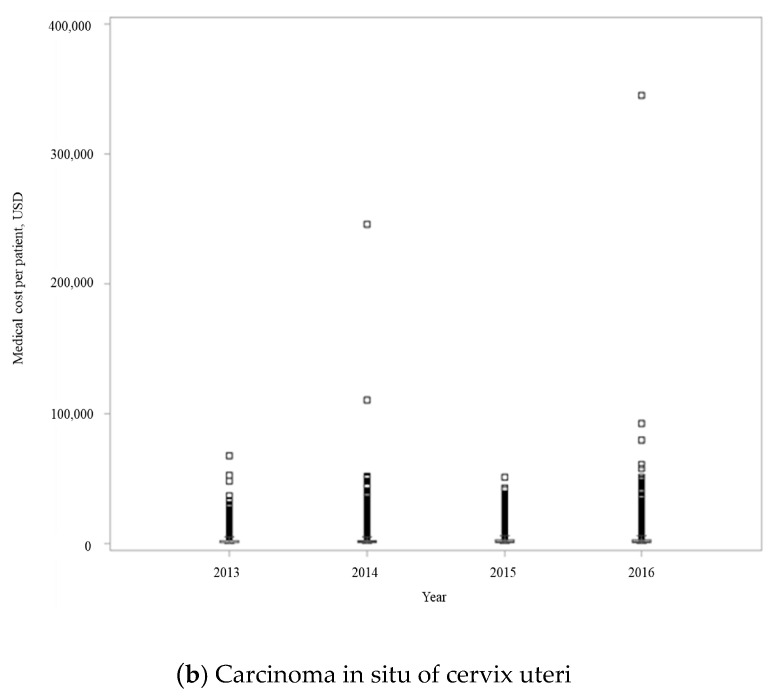
Incidence-based medical cost per patient with cervical cancer and CIS by year.

During the study period, the number of patients diagnosed with cervical cancer and CIS were 15,616 and 27,772, respectively. The crude incidence rate of cervical cancer decreased from 19.7 per 100,000 females in 2013 to 18.9 per 100,000 people in 2016, while that of CIS increased from 30.9 per 100,000 people in 2013 to 38.2 per 100,000 people in 2016 (data not shown). Table 1 shows the number of patients and the medical cost of cervical cancer and CIS from 2013 to 2016. The distribution of medical costs for each disease from 2013 to 2016 is presented as a boxplot (Figure A1). As of 2016, the proportion of patients aged 40–59 (1933 out of 3971, 48.7%) was the largest in cervical cancer patients, followed by those over 60 (1264 out of 3971, 31.8%). However, the highest proportion of patients with CIS was in those aged 20–39 years (3485 out of 8029, 43.4%), followed by those aged 40–59 (3434 out of 8029, 42.8%). Therefore, the proportion of cervical cancer was highest among older patients, while CIS mainly occurred in younger patients. In both patient groups, the 20–39 year old patients had the lowest medical costs during the study period. With regard to cervical cancer patients, the mean medical cost was similar in the age groups >40 years; however, the medical cost of both patient groups was statistically different according to age in each year (both parametric and nonparametric test, *p* < 0.0001). Not surprisingly, the medical cost of both patient groups increased significantly with age and more comorbidities (both parametric and nonparametric tests, *p* < 0.0001).

### 3.2. Age-Specific Incidence Rate

**Figure 2:**
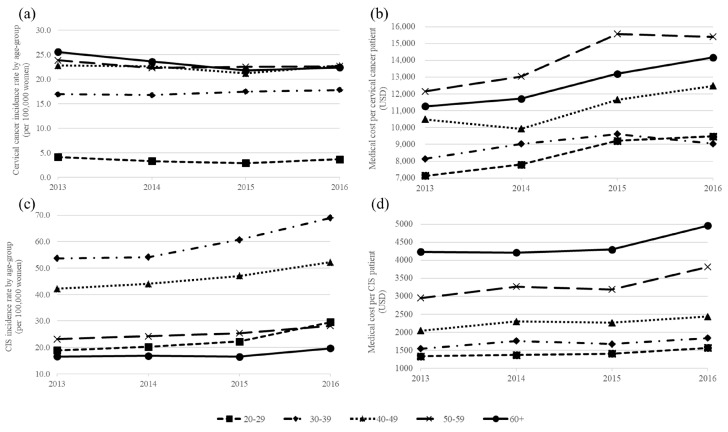
Age-specific incidence rate and mean medical costs of cervical cancer and CIS by year. (**a**) The age-specific incidence rate of cervical cancer was calculated per 100,000 people. (**b**) The mean medical cost of cervical cancer was adjusted by the 2016 medical care component CPI and converted to US dollars. (**c**) The age-specific incidence rate of CIS was calculated per 100,000 people. (**d**) The mean medical cost of CIS was adjusted by the 2016 medical care component of CPI and converted to US dollars.

More detailed age-specific incidence rates and medical costs by age groups are presented in Figure 2. Figure 2a shows the age-specific incidence rate of cervical cancer from 2013 to 2016. Among all age groups, the incidence rate was the lowest in 20–29 year olds, while the highest rate was in those >60 years old except in 2016. Over time, the incidence tended to decrease in most age groups, but increased from 16.9 per 100,000 in 2013 to 17.8 per 100,000 in 2016 for those aged 30–39. The age-specific incidence rate of CIS from 2013 to 2016 is presented in Figure 2c. The incidence rate of all age groups increased. The incidence rate of 30–39 year olds was the highest, while that in those over 60 was the lowest. In particular, the incidence of patients in their 20 s and 30 s increased significantly over time compared to that of other age groups. The incidence in 20–29 year old patients increased 56.8% from 18.9 per 100,000 in 2013 to 29.6 per 100,000 in 2016. The incidence in those aged 30–39 years increased 28.4% from 53.7 per 100,000 in 2013 to 69 per 100,000 in 2016.

### 3.3. Incidence-Based Medical Cost by Age Groups

In Figure 2b,d, the mean medical costs of cervical cancer and CIS are presented from 2013 to 2016. The medical costs for all age groups of both patient groups increased in 2016 compared to those in 2013. As of 2016, the mean medical cost of cervical cancer was the highest (at USD 15,400) for those aged 50–59, while the mean medical cost of CIS was highest (at USD 4959) for patients aged >60 years. The medical cost of CIS patients increases with age.

### 3.4. Regression Analysis

In order to adjust for various factors associated with the cost of the cervical cancer and CIS, the LRM with log-transformation and GLM with gamma distribution were applied (Table 2). In the univariate analysis, all variables were significantly associated with the cost of both patient groups (*p* < 0.0001). In the multivariate analysis, the LRM showed a better fit than the GLM, since the AIC score was smaller. For both patient groups, the age, year, and CCI variables had positive impacts on the medical cost. For instance, the medical costs increased in older patients and those with large CCI scores. For example, the effect increased with age as compared to that of the group aged 20–39. The cost difference by age according to the regression coefficient for cervical cancer was USD 6400 (Exp(8.263115 + 0.50095)) for 40–59 year olds, and USD 7005 (Exp(8.263115 + 0.59136)) for patients aged >60. In the case of CIS, the cost was USD 1230 (Exp(6.85153 + 0.26326)) and USD 2070 (Exp(6.85153 + 0.78204)) in those aged 40–59 and >60 years, respectively.

## 4. Discussion

We analyzed the crude incidence rate and incidence-based cost of cervical cancer and CIS from 2013 to 2016 from the HIRA database. As in previous studies in Korea [14], we found that the crude incidence rate of cervical cancer decreased over time, while that of CIS increased during the study period. The age-specific incidence of cervical cancer was similar in those aged >40 years, while the age-specific incidence of CIS peaked at 30–39 years of age. This result was comparable to that reported in studies regarding the predominant age of CIS [4,30].

The number of new patients in our study is somewhat higher than the results of a previous study [11], which could be attributable to incompleteness and underestimated limitations of the cancer registration data. In our study, the crude incidence rate of cervical cancer was 18.8 per 100,000 people in 2014. According to a study conducted in Korea, the incidence rate of cervical cancer was 28.4 per 100,000 people in 2014 [19]. The overall incidence rate of a previous study was higher than that in our study due to differences in study design, such as the washout period. However, the decreasing incidence of cervical cancer is consistent with our findings.

The trend of increasing incidence of CIS in patients in their 20 s and 30 s has been shown in previous studies in the US and Netherlands, as well as in Korea [12,13,14]. It is unclear whether this increase is related to increased exposure to risk factors or active cervical cancer screening [31]. However, recent changes in sexual behavior observed in previous studies in Korea and an increase in the age of cervical cancer screening may affect this trend. The proportion of abnormal cytological test results is higher in younger women than in older women. These data suggest that young women have different sexual behavior than older women [32]. The average age of first sexual intercourse has also decreased. According to results of the 2012 Korea Youth Risk Behavior Web-based Survey, the age of first sexual intercourse decreased from 13.9 years in 2008 to 13.2 years in 2015 [33]. Another previous study in Korea that surveyed 2400 women aged 12–29 years showed that approximately 39% of women have already had sexual intercourse [34]. In addition, the Korean government expanded the target population of cervical cancer screening to women aged >20 in 2016 [35]. Our study cannot directly analyze the effect of delaying the target age on the incidence of CIS and cervical cancer. However, following the result of our study, the incidence of CIS in patients aged 20–29 years in 2016 increased by approximately 33% compared to that in 2015.

The medical costs of cervical cancer and CIS increased during the study period. In particular, the medical cost of cervical cancer patients was the highest in the group aged 50–59, and the next highest in those over 60. These results were in contrast to CIS, which tends to cost more with a higher age. In a previous study in Korea, the proportion of cervical cancer patients over 65 years old who had advanced staged disease was 51%, which is higher than that of patients under 65 years of age (24.4%) [4]. In the same context, patients over 65 tended to be diagnosed with advanced stages of disease and the proportion of nontreatment was relatively high [36]. Therefore, the treatment cost of older patients may be lower than that in patients in their 50 s (which is the predominant incidence group). The medical cost of CIS increased with increasing age. This finding was consistent with that of previous study findings, which showed that older patients had higher medical costs because they had more comorbidities and higher disease severity [19]. The data in this study also showed the highest proportion of patients in their 60 s with a CCI score of 2 or 3 (29.3% and 53.2%, respectively).

Nevertheless, the cost estimations from other studies are not directly comparable due to the prevalence-based cost or differences in study design. All studies in Korea consistently report that the burden of cervical cancer was constantly increasing [17,18,19]. One previous study [18] estimated the prevalence-based cost of HPV-associated diseases in Korea using claims data in 2015, and suggested that the cost of cervical cancer per patient was USD 2726. These results are nearly five times lower than ours (USD 12,688 in 2015). The prevalence-based cost estimates the 1 year cost of all patients with the disease. However, the incidence-based cost has the advantage of estimating the cost of newly diagnosed patients and can reflect factors that directly influence the cost of care [37,38].

The regression analysis demonstrated that the treatment cost was higher in older age groups than it was in the 20–39 years old group. This result suggests that the more the disease can be detected through cervical cancer screening in young women, the more treatment costs can be reduced. In a study conducted in the UK, the lower the International Federation of Gynecology and Obstetrics (FIGO) stage of cervical cancer, the higher the incidence of patients in their 20 s (compared to in their 30 s) [31]. A similar Chinese study found that the number of patients corresponding to FIGO stage I, the lowest stage, was higher in patients aged <65 years than it was in those aged >65 [36]. These findings suggest that the younger the patients, the lower the severity of the disease. In addition, in one previous study undertaken in the UK, the stage-specific treatment cost of cervical cancer for stage 1 was significantly lower than that for stages 2–4 and the treatment cost [39]. Therefore, patients of younger age are more likely to be diagnosed at an early stage through screening, and the treatment cost is lower.

This study has several limitations. First, the NHI claims data are used to process the reimbursement. Therefore, the nonreimbursed services and indirect costs, such as transportation costs, caregiver costs, and lost productivity, are not included. Additionally, the data information such as the disease severity or stage of disease is not provided in NHI claims data [21]. Therefore, the burden of the diseases may have been underestimated. Second, the operational definition of patients was based on the primary or secondary diagnosis. Therefore, our estimates may vary depending on the diseases recorded after the secondary diagnosis. In addition, the washout period and follow-up period might affect the estimation. Despite these limitations, our study is an incidence-based study using a large database of HIRA claims data covering 98% of the population in Korea [21]. In addition, the operational definition was fully discussed through the design of prior studies [18,19] and consultation with clinicians.

Our results suggest that the costs of both cervical cancer and CIS are associated with a patient’s age, even after adjusting for confounding factors. These results suggest that early detection through cervical cancer screening in young women can alleviate the burden of cervical cancer. Even when cervical cancer is detected at an earlier stage through screening, there are benefits with life expectancy (LF) and expected years of life lost (EYLL). Younger patients have more life-related benefits than do older patients [40]. These results suggest that the benefit of cervical cancer screening is greater in young women than it is in older women.

In addition to screening, HPV vaccination is a possible option for cervical cancer prevention. Currently, HPV vaccines have been included in the NIP (National Immunizations Program) since 2016 for 12 year old girls in Korea [41]. However, HPV vaccination prior to sexual behavior (<13 years) in both males as well as females could bring enhanced public health benefit [42]. Therefore, HPV vaccination for boys aged under 13 could be an option for cervical cancer prevention as well.

However, over the 10 year period, cervical cancer screening participation rate increased by 0.9% annually, which was the lowest increase among five major cancers including gastric cancer, colorectal cancer, liver cancer, breast cancer, and cervical cancer [43]. The reasons for noncompliance with recommended cervical cancer screening include: a fear of being diagnosed with cancer, pain or discomfort during the screening procedure, and lack of knowledge about the screening [44]. One study that surveyed Korean women in their 20 s found that only 36.5% knew that the cervical cancer screening age had expanded [45]. Therefore, the Korean government should increase its educational approaches to improve the public’s awareness of the screening recommendations.

## 5. Conclusions

Our study estimated the incidence-based treatment cost of cervical cancer and CIS patients using the latest available, nationally representative data. The crude incidence rate of cervical cancer is decreasing. However, the incidence rate of CIS is increasing in women, and especially in those in their 20 s and 30 s. The regression results demonstrated that the treatment cost was lower in younger women than it was in older women. These results suggest that early detection by regular screening in younger women is cost effective with regard to HPV-related cervical diseases in Korean society.

## Figures and Tables

**Table 1:** The number of patients and distribution of cervical cancer and cervix uteri (CIS) between 2013 and 2016.

Variable			Cervical Cancer					CIS				
			*N* (%)	Mean (±SD)	*p* Value ^1^	Median	*p* Value ^2^	*N* (%)	Mean (±SD)	*p* Value ^1^	Median	*p* Value ^2^
**2013**	**Age, years**	20–39	795 (19.8)	7979 (±9980)	<0.0001	5049	<0.0001	2699 (43.0)	1499 (±2166)	<0.0001	917	<0.0001
		40–59	1949 (48.7)	11,303 (±10,413)		8850		2767 (44.0)	2347 (±3354)		1338	
		60+	1259 (31.5)	11,273 (±9473)		9759		816 (13.0)	4235 (±5145)		2777	
	**CCI**	0	2796 (69.8)	10,311 (±9780)	0.012	7667	0.021	5064 (80.6)	1876 (±2580)	<0.0001	1039	<0.0001
		1	556 (13.9)	11,071 (±10,973)		8845		654 (10.4)	2885 (±3719)		1812	
		2	453 (11.3)	11,458 (±10,477)		9293		434 (6.9)	4165 (±6177)		2295	
		3	198 (5.0)	12,078 (±11,324)		8830		130 (2.1)	6167 (±6994)		3378	
**2014**	**Age, years**	20–39	746 (19.3)	8864 (±10,621)	<0.0001	5294	<0.0001	2709 (41.7)	1669 (±3459)	<0.0001	976	<0.0001
		40–59	1905 (49.3)	11,421 (±11,248)		8067		2919 (45.0)	2629 (±6111)		1386	
		60+	1213 (31.4)	11,719 (±10,430)		9448		864 (13.3)	4214 (±5231)		2707	
	**CCI**	0	2743 (71.0)	10,532 (±10,138)	0.000	7422	0.002	5254 (80.9)	2014 (±4385)	<0.0001	1120	<0.0001
		1	467 (12.1)	12,388 (±13,063)		8893		655 (10.1)	2931 (±3568)		1891	
		2	429 (11.1)	11,912 (±12,161)		7997		449 (6.9)	5330 (±9653)		2044	
		3	225 (5.8)	12,437 (±12,350)		8937		134 (2.1)	7041 (±8938)		3574	
**2015**	**Age, years**	20–39	749 (19.8)	9570 (±12,592)	<0.0001	5227	<0.0001	2983 (42.8)	1613 (±2353)	<0.0001	1042	<0.0001
		40–59	1854 (49.1)	13,624 (±13,843)		8876		3095 (44.4)	2580 (±3845)		1397	
		60+	1175 (31.1)	13,198 (±11,861)		10,840		891 (12.8)	4296 (±5464)		2892	
	**CCI**	0	2703 (71.5)	12,646 (±13,131)	0.02	8216	0.008	5711 (81.9)	2032 (±2894)	<0.0001	1170	<0.0001
		1	445 (11.8)	12,272 (±11,380)		8639		701 (10.1)	2789 (±3566)		1765	
		2	411 (10.9)	13,295 (±13,683)		8849		422 (6.1)	5008 (±7111)		2362	
		3	219 (5.8)	15,152 (±14,562)		10,383		135 (1.9)	7050 (±8318)		3709	
**2016**	**Age, years**	20–39	774 (19.5)	9110 (±11,368)	<0.0001	5179	<0.0001	3485 (43.4)	1767 (±2745)	<0.0001	1145	<0.0001
		40–59	1933 (48.7)	13,909 (±14,979)		8822		3434 (42.8)	2906 (±7456)		1469	
		60+	1264 (31.8)	14,176 (±12,932)		10,753		1110 (13.8)	4959 (±6135)		3249	
	**CCI**	0	2745 (69.1)	12,486 (±13,530)	0.000	7771	<0.0001	6550 (81.6)	2230 (±5249)	<0.0001	1263	<0.0001
		1	548 (13.8)	14,450 (±14,456)		10,101		795 (9.9)	3463 (±4811)		2118	
		2	441 (11.1)	13,408 (±13,100)		9586		538 (6.7)	5955 (±8630)		3077	
		3	237 (6.0)	15,822 (±16,430)		10,735		146 (1.8)	7525 (±11,102)		3739	

CIS, carcinoma in situ of cervix uteri; SD, standard deviation; CCI, Charlson Comorbidity Index. Unit: US dollars. ^1^ Estimated based on the ANOVA test. ^2^ Estimated based on the Kruskal–Wallis test.

**Table 2:** Linear regression model and generalized linear model for incidence-based medical costs.

Model	Variable	Cervical Cancer (C53)				CIS (D06)			
		Univariate		Multivariate		Univariate		Multivariate	
		*β*	*p* Value	*β*	*p* Value	*β*	*p* Value	*β*	*p* Value
Linear Regression Model	**Age** (reference = 20–39 years)								
	40–59	0.509	<0.0001	0.501	<0.0001	0.306	<0.0001	0.263	<0.0001
	60+	0.617	<0.0001	0.591	<0.0001	0.95	<0.0001	0.782	<0.0001
	**Year** (reference = 2013)								
	2014	0.036	0.219	0.035	0.223	0.049	0.007	0.045	0.008
	2015	0.124	<0.0001	0.129	<0.0001	0.077	<0.0001	0.084	<0.0001
	2016	0.13	<0.0001	0.131	<0.0001	0.184	<0.0001	0.186	<0.0001
	**CCI** (reference = 0)								
	1	0.164	<0.0001	0.118	0.000	0.42	<0.0001	0.27	<0.0001
	2	0.131	<0.0001	0.049	0.134	0.744	<0.0001	0.571	<0.0001
	3	0.278	<0.0001	0.17	0.000	1.149	<0.0001	0.827	<0.0001
	AIC			500,560				788,947	
Generalized Linear Model	**Age** (reference = 20–39 years)								
	40–59	0.348	<0.0001	0.335	<0.0001	0.469	<0.0001	0.398	<0.0001
	60+	0.35	<0.0001	0.321	<0.0001	0.998	<0.0001	0.821	<0.0001
	**Year** (reference = 2013)								
	2014	0.036	0.12	0.038	0.093	0.09	<0.0001	0.076	<0.0001
	2015	0.177	<0.0001	0.177	<0.0001	0.068	<0.0001	0.076	<0.0001
	2016	0.205	<0.0001	0.198	<0.0001	0.19	<0.0001	0.181	<0.0001
	**CCI** (reference = 0)								
	1	0.093	0.000	0.059	0.001	0.39	<0.0001	0.22	<0.0001
	2	0.089	0.001	0.046	0.014	0.924	<0.0001	0.785	<0.0001
	3	0.198	<0.0001	0.134	<0.0001	1.223	<0.0001	0.968	<0.0001
	AIC			542,775				872,825	

CIS, carcinoma in situ of cervix uteri; CCI, Charlson Comorbidity Index; AIC, Akaike Information Criterion.
